# Pattern of skin diseases in children attending a dermatology clinic in a referral hospital in Wolaita Sodo, southern Ethiopia

**DOI:** 10.1186/s12895-019-0085-5

**Published:** 2019-04-08

**Authors:** Abraham Getachew Kelbore, Philip Owiti, Anthony J. Reid, Efa Ambaw Bogino, Lantesil Wondewosen, Blen Kassahun Dessu

**Affiliations:** 1 College of Health Sciences and Medicine, Dermatology Department, Wolaita Sodo University, Wolaita Sodo, Ethiopia; 2 International Union Against Tuberculosis and Lung Disease (The Union), Paris, France; 3 The National Tuberculosis, Leprosy and Lung Disease Programme, Ministry of Health, Nairobi, Kenya; 4 Operational Research Unit, MedicalDepartment, Operational Centre Brussels, Médecins Sans Frontières, LuxOR, Luxembourg City, Luxembourg; 5 College of Health Sciences and Medicine, Wolaita Sodo University, Wolaita Sodo, Ethiopia

**Keywords:** Pediatrics, Skin diseases, Hospital-based, Epidemiological study, SORT IT, Ethiopia

## Abstract

**Background:**

Epidemiological studies to determine the pattern of skin diseases among children are important for proper health care planning and management. The purpose of this study was to describe the pattern of skin diseases among pediatric patients seen at a dermatology outpatient clinic of Wolaita Sodo Teaching and Referral Hospital, southern Ethiopia.

**Method:**

We conducted a retrospective hospital-based, cross-sectional study between January 2016 and December 2017 at a teaching and referral hospital dermatology outpatient department. All children younger than 15 years presenting with newly-diagnosed skin diseases were included. Diagnosis was mainly made clinically, with some laboratory support.

**Results:**

A total of 1704 children with 1869 new skin diagnoses were included, of whom 139 (8.2%) had more than one disease. Of the children, 52.4% were males and 44.9% within the age-group 5-10 years. Eczematous dermatitis accounted for the largest group (23.9%, *n* = 447) of skin conditions followed by bacterial infections (21.3%, *n* = 398), fungal infections (18.8%, *n* = 351) and infestations (9.9%, *n* = 185). Seasonal variation was demonstrated, with eczematous conditions and bacterial infections being higher during autumn and winter.

**Conclusion:**

Overall, eczema, bacterial and fungal infections were the three major pediatric skin diseases occurring among children attending this hospital’s outpatient department. There was seasonal variation in some of the skin diseases. This study gives a snapshot of skin disorders presenting to hospital in children in southern Ethiopia and may help to plan dermatology service expansion, educational programs and preventive measures.

## Background

World-wide, skin diseases have stimulated a lot of interest over the years because they are common but potentially preventable and controllable [1]. In 2013, skin diseases were the 18th leading cause of global disability-adjusted life years (DALYs) and the fourth leading cause of non-fatal disease burden worldwide [2]. In Africa, skin diseases are estimated to affect between 21 and 87% of children and are the reason for up to a third of outpatient visits to pediatricians and dermatologists [3]. While skin diseases have low mortality, and thus are given less attention than more serious diseases, [4, 5] their contribution to overall morbidity causes a significant burden to the community, placing a strain on health care services’ finances and personnel [1].

The pattern of skin diseases varies from country to country and even from region to region within the same country due to ecological factors, hygienic standards, social customs and genetics [6, 7]. In developed countries, eczematous skin diseases are the most common among children [3], whereas in most developing countries infections and infestations are predominant [4, 8]. For instance, studies in Nigeria, Bangladesh, India, Brazil, Tanzania and Egypt showed different patterns of skin diseases by country among school children with infections most common overall [4–7, 9].

In Ethiopia, there is very limited information on the profile of skin diseases in children. In a cross-sectional, hospital-based study in rural Ethiopia the most common skin diagnoses in children under five were infestations like scabies and pediculosis, pyoderma, fungal infections and eczema [10]. However, this study had a small sample size and was limited to 3 months duration. A retrospective study in a large urban referral hospital in northern Ethiopia found eczema to be most common diagnosis but it included adult patients [11]. A community survey in south-west Ethiopia was limited in scope but found parasitic infestations most common [12]. Finally, a community survey and a mobile clinic in central Ethiopia revealed infectious diseases as most important [13]. These studies provide some information but they were of short duration (weeks to months) and had smaller sample sizes.

There are no data regarding patterns of skin diseases among children in southern Ethiopia. Given the variation in study designs, sample sizes and the effects of different geographical and socio-economic situations, we wanted to document the pattern of skin diseases in a pediatric population seen at the dermatology department of a referral hospital in southern Ethiopia over a longer period and with greater numbers than previously. In addition, no study has examined the effect of the three climatic seasons in Ethiopia on the patterns of skin presentations.

We thus report on, among pediatric patients seen at the dermatology outpatient department of Wolaita Sodo Teaching and Referral Hospital, southern Ethiopia from January 2016–December 2017: 1) the demographic characteristics of patients attending the clinic, 2) the frequency and proportion of dermatology diagnoses, 3) the seasonal variation of the five most common groups of dermatologic conditions according to the three main seasons: summer, autumn, and winter, and 4) the association between the demographic characteristics and diagnostic categories of the skin conditions.

## Method

### Study design

This was a descriptive cross-sectional study using retrospectively collected routine hospital data.

### General setting

Ethiopia is located in the Horn of Africa and is the third most populous country in sub-Saharan Africa with more than 100million inhabitants, of whom more than 80% live in rural areas. The country has approximately 80 different ethnic groups and is considered a lower-middle-income country with a GDP per capita of USD706 per annum. Health service coverage is estimated at approximately 64%, with the majority of the population served through primary health care facilities [14]. According to the Ethiopian National Meteorological Services Agency (NMSA) Ethiopia has three seasons based on the average trends of the weather and rainfall: *Kiremt* (summer) –between the months of June and September, experiencing long and heavy rainfall;*Bega* (Winter) - between October and January, the dry season; and *Belg* (Autumn) - between February and May – experiencing short and moderate rainfall. The altitude of the Wolaita zone ranges from 501 to 2738 m above sea level. The annual average temperature of the zone is 21.9 °C and there are two seasonal variations only for temperature.

### Study area

The present study was conducted in Wolaita Sodo Teaching and Referral Hospital, which is the only hospital out of six (two general and four primary hospitals in its catchment area) providing dermatology services in Wolaita Sodo town, in the southern part of Ethiopia since 2000. Currently, the hospital provides various clinical and referral services for approximately two million patients ranging from primary to specialized care and serves patients referred from different health facilities in the region. It has an average bed capacity of 200 and about 250 health professionals including specialists, tropical dermatology professionals, general practitioners, nurses, midwives, pharmacists and others. The hospital investigation capacity includes laboratory, radiography and ultrasound services [15].

### Diagnosis and management of skin diseases

All children who presented with skin conditions were managed by three professionals, two BSc nurses trained in Masters of Tropical Dermatology and one dermatologist, the latter who joined in mid-2017, at the dermatology outpatient unit. Inpatient and other consultation services for dermatology problems were also provided in the facility. Children who had skin problems were screened at a central triage area after which they were scheduled for dermatology consultation. The diagnoses were mainly made clinically, but relevant laboratory investigations or histopathology were performed when the diagnosis was unclear. They included laboratory investigations like the KOH test, skin smear tests and gram stain or histopathology. Children above 2 years of age were eligible for HIV testing.

### Study population and period

All children younger than 15 years who had been newly diagnosed with a skin disease at the dermatology outpatient clinic of Wolaita Sodo Teaching and Referral Hospital, southern Ethiopia between January 2016 and December 2017 were included.

### Data variables, data collection and sources of data

The study variables included the children’s demographic characteristics (age, gender and residence), HIV status, diagnoses of skin diseases and date of diagnosis: bacterial infections, eczema, fungal infections, viral Infections, pigmentary diseases, parasite infestations, drug eruption, protozoal infections, papulosquamous diseases, pilosebaceous diseases and genodermatosis. Ethiopian seasonal variables are: summer, autumn and winter.

The source of data was the hospital dermatology outpatient register. A uniform data abstraction sheet was prepared to collect the relevant data from the registers. Data were collected by two trained tropical dermatology professionals from the dermatology outpatient registers between May and July 2018.

### Analysis

Data was double-entered from the paper-based abstraction sheets into EpiData software for data entry and analysis *(v4.2.0.0 for entry and v2.2.2.182 for analysis, EpiData Association, Odense, Denmark).* The diagnoses were grouped into their classes: bacterial, eczema, fungal, infestation and dates of diagnoses were used to define the seasons. A descriptive analysis of the children’s characteristics, disease conditions and seasonal variations was performed and presented in frequencies and proportions. An association between the disease conditions and seasonal patterns was ascertained by using the Chi-square test. Level of significance was set at 5%.

## Results

### Socio-demographics characteristics

There were 1704 pediatric patients newly-diagnosed and treated at the dermatology outpatient department from January 2016 to December 2017. Their socio-demographic characteristics and HIV status are shown in Table 1. There were slightly more males than females and most patients came from the surrounding urban context. Very few eligible patients had HIV testing.

**Table 1:**
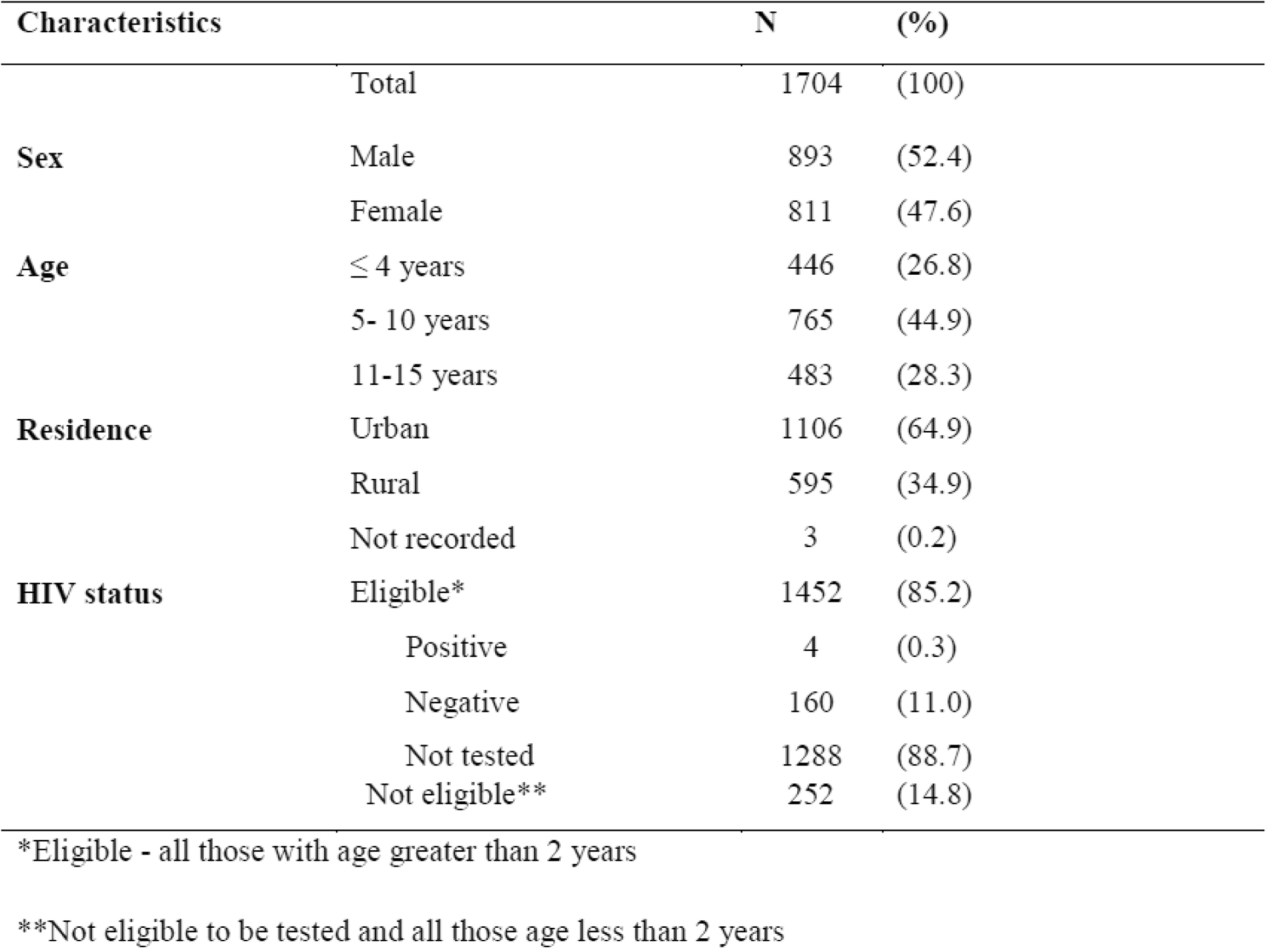
Demographic characteristic of pediatic patients attending dermatology department at Wolaita Sodo Teaching and Referral Hospital in Southern Ethiopia, January 2016-December 2017

### Pattern of pediatric skin diseases

The numbers of diseases diagnosed among the 1704 children in the dermatology outpatient department are presented in Table 2. The five most common categories of skin diseaseswere eczema (23.9%), bacterial infections (21.3%), fungal infections (18.8%), infestations (9.9%), and pigmentary diseases (7.4%). Regarding individual diagnoses, impetigo was the most frequently presenting skin disease (13.8%) followed by tinea capitis (12.7%), atopic dermatitis (11.3%), and scabies (9.6%). One case of podoconiosis and one of folliculitis deculvans were also identified and merged as “other” in the category of miscellaneous diseases. The total number of skin diseases diagnosed was greater than the number of patients because 139 children had more than one disease.

**Table 2:**
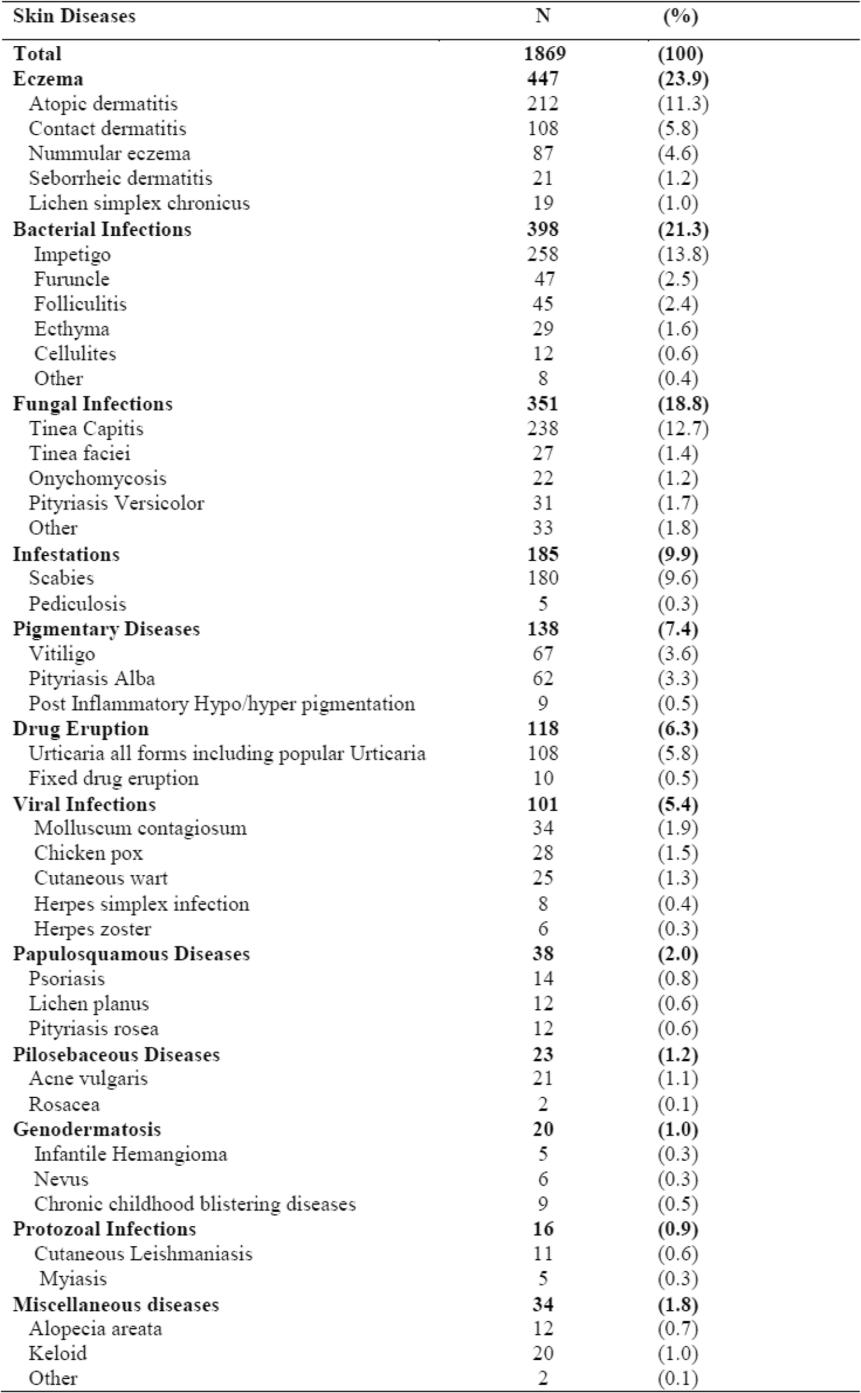
Frequencies and proportion of skin diseases among pediatric patients attending dermatology department at Wolaita Sodo Teaching and Referral Hospital in Southern Ethiopia, January 2016-December 2017

**Fig. 1:**
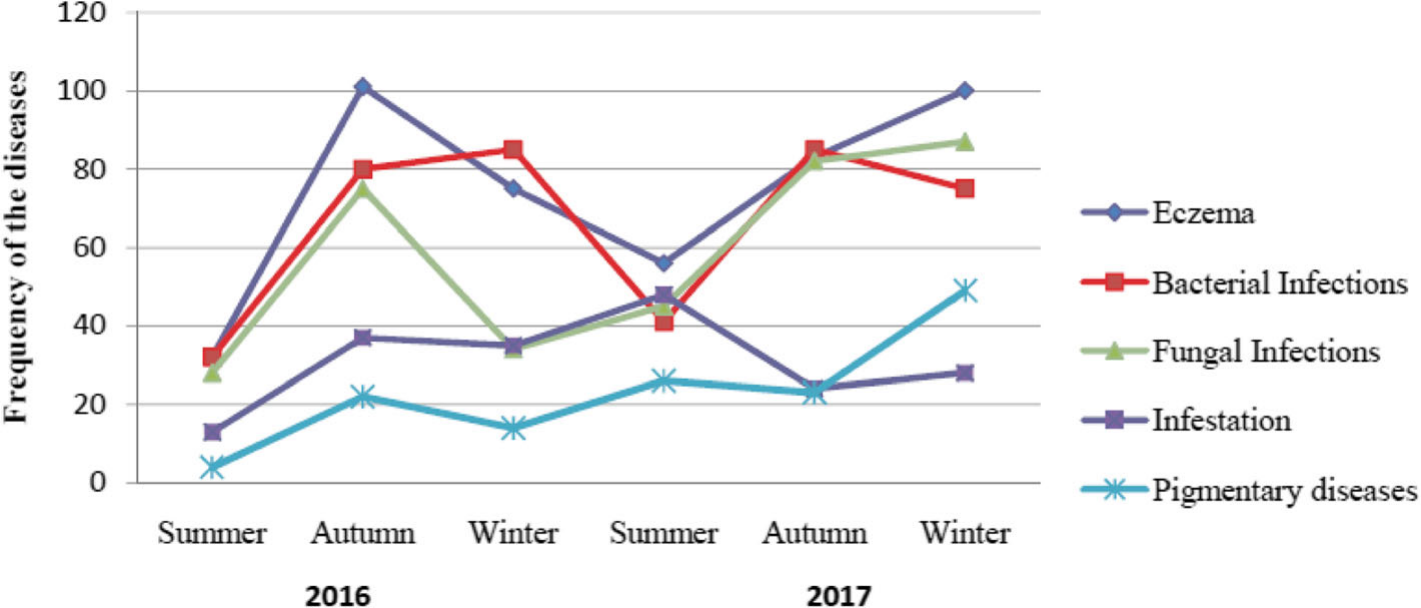
Seasonal trend of the top five skin diseases among the pediatric patients attending dermatology department at Wolaita Sodo Teaching and Referral Hospital, Ethiopia, January 2016-December 2017

Figure 1 shows the seasonal variation of the top five diagnoses during the study period. Generally, the diseases increased during autumn and winter but with wide variations between them.

Table 3 shows the distribution of the top five diagnoses by gender during the study period. Eczema and infestations were more prevalent among men (58 and 63% respectively).

**Table 3:**
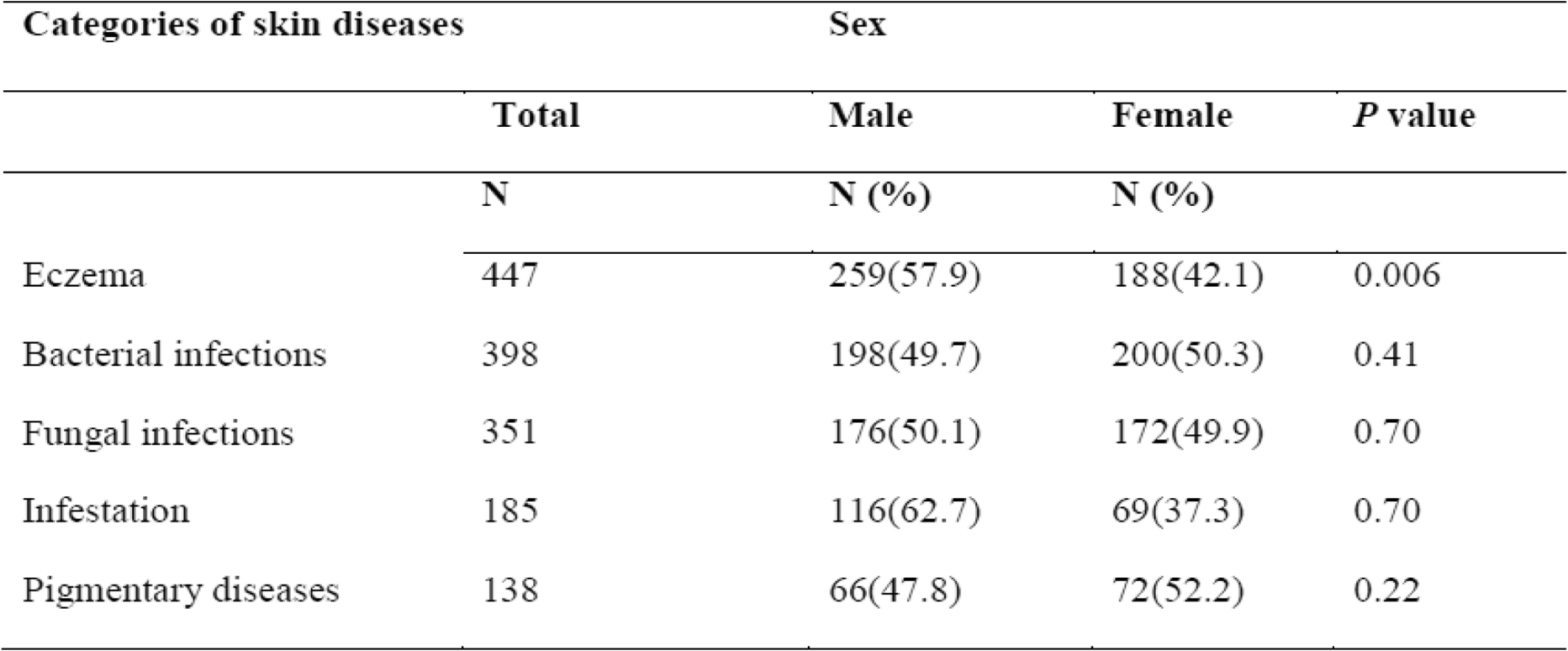
Gender distribution of the five most commom skin diseases categories among pediatric patients attending dermatology depatment at Wolaita Sodo Teaching and Referral Hospital in Southern Ethiopia, January 2016-December 2017

The five most skin diseases categories were more common among children residing in urban contexts as compared to rural. However, there was no statistical difference between urban and rural residence for the most common skin diseases categories (Table 4).

**Table 4:**
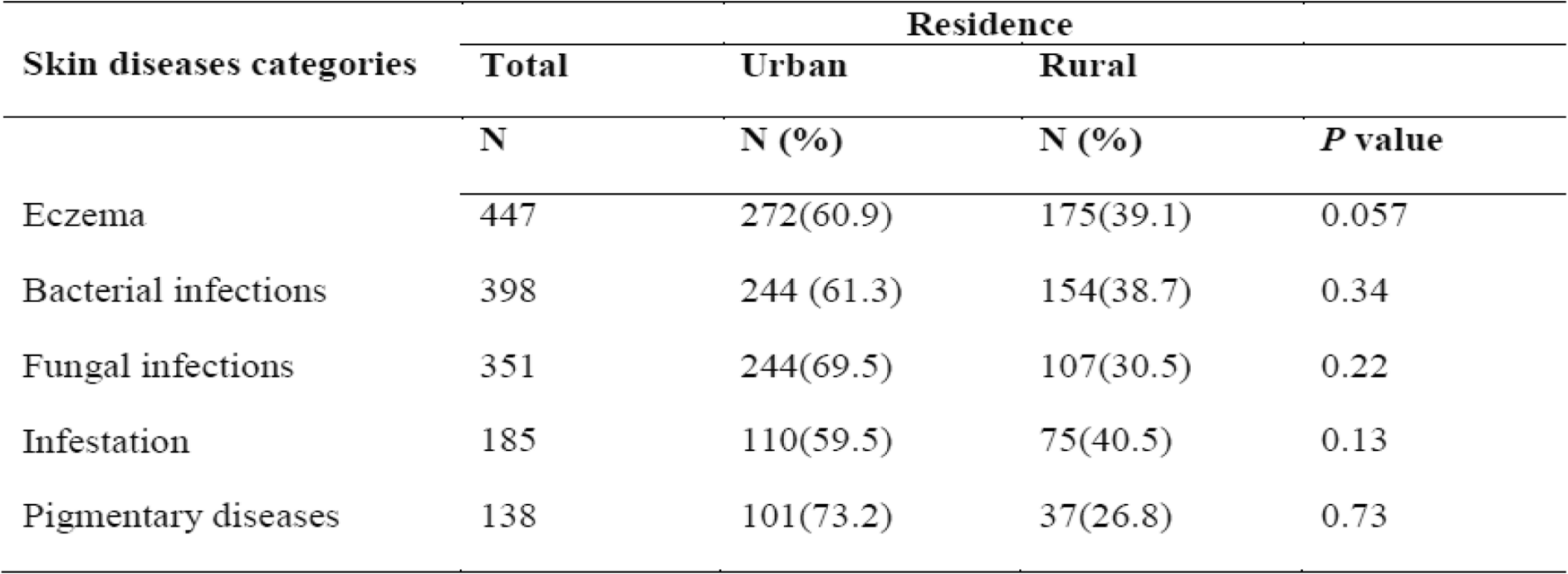
Distribution of the most five commom Skin diseases category with residence among padiatric patients attending dermatology department at Wolaita Sodo Teaching and Referral Hospital in Southern Ethiopia, January 2016-December 2017

## Discussion

This is the first study of skin diseases in children in southern Ethiopia over a 2 year period. It shows that eczematous diseases, which include atopic dermatitis, nummular eczema, contact dermatitis, seborrheic dermatitis and lichen simplex chronicus, were the most common followed by bacterial infections, fungal infections, infestation and pigmentary diseases. This pattern seems similar to those of studies conducted in Mekelle, Addis Ababa, Pakistan and western Nepal [11, 16–18].

However, the pattern of skin diseases is different from those described in other studies in south- west Ethiopia, Nigeria, Egypt and Turkey [10, 19–21]. Parasitic infestation, fungal and bacterial infections were prominent in these studies. The differences may be due to differences in geographical location, sample size, duration of the study, study design, different patient age limits, and socio-economic status. In particular, the other studies were based on out-patient populations, which were different from our hospital-based one and this may explain some differences.

Our study also identified a higher number of bacterial infections and fungal infections followed with viral infections like chicken pox, cutaneous wart and molluscum contagiosum. This is consistent with studies done in other parts of Ethiopia and Tanzania [13, 22] which suggest that a hot and humid climate increases susceptibility to infectious skin diseases. Poor sanitation and low socio-economic status of the patients may also be a factor. The magnitude of scabies was 9.6% in our study which was similar to another study conducted in southern Ethiopia [12], but it is different from a study conducted in Nepal which showed a 4.4%rate [18]. Poor hygiene in winters along with poor sanitation may be the cause of increased prevalence of scabies in developing countries.

This study identified seasonal variation in the pattern of skin diseases. Eczema, bacterial and fungal infections were increased during the autumn and winter seasons. Although we couldn’t identify a study in Ethiopia to compare, seasonal variation in certain skin disorders is a well-known phenomenon that has been observed [23]. With changes in seasons there can be variation in temperature, humidity, ultraviolet rays, wind, atmospheric pollen allergens, and humidity that can have an impact on epidermal barrier function [24].

The children diagnosed with skin diseases in this study were mostly from the urban context surrounding the hospital, (65%) which is consistent with a study done in another part of Ethiopia [12]. This undoubtedly reflects easier access to health care. Our study also reported a few emerging neglected tropical diseases such as cutaneous leishmaniasis and a rare skin disease--chronic bullous disease of childhood. This study highlights the potential need for more dermatology training, service expansion at primary health care level and management skills of common childhood skin diseases.

The strengths of this study include the large sample size, a longer study duration (2 years), and standardization of diagnoses, given that they were made by a limited number of dermatology professionals. However, it was hospital-based and, as such, is not generalizable to the community. As well, milder skin conditions are usually treated in primary care centers and the profile of diagnoses in the hospital would not reflect them. Likewise, with the diagnoses mostly being clinical, there may have been some element of subjectivity. However, the three dermatology staff consulted frequently amongst themselves, mitigating these effects. Lastly, being retrospective in nature we were unable to collect socio-economic aspects of the patients which are important factors to understanding skin diseases.

## Conclusion

Overall, eczema, bacterial and fungal infections were the three most common pediatric skin diseases seen in the outpatient dermatology clinic in a teaching hospital in southern Ethiopia. There were some seasonal variation in some diseases. The pattern of pediatric skin disorders represents the distribution of skin diseases in children seen at an outpatient hospital department in Wolaita Sodo and provides a basis for future health planning. Monitoring the epidemiology of skin disorders in children may help to plan dermatology service expansion within the region, educational programs and preventive measures.

## Abbreviations

BSc: Bachelor of Science
DALYs: Global disability-adjusted life years
GDP: Gross Domestic Product
HIV: Human Immunodeficiency virus
KOH: Potassium Hydroxide
NMSA: National Meteorological Services Agency
SORT IT: Structured Operational Research and Training Initiative
USD: United State Dollar
